# Obesity-Related Inflammatory Biomarkers in the Elderly Population

**DOI:** 10.3390/cells14211733

**Published:** 2025-11-04

**Authors:** Georgia Vamvakou, Nikolaos Theodorakis, Dimitris Anagnostou, Magdalini Kreouzi, Loukianos S. Rallidis, Vasiliki Katsi, Effie Simou, Stefanos Archontakis, George Skalis, Christos Hitas, Konstantinos Toutouzas, Maria Nikolaou

**Affiliations:** 1 Department of Cardiology, Amalia Flemig General Hospital, 15127 Melissia, Greece; n.theodorakis@flemig-hospital.gr (N.T.); d.anagnostou@flemig-hospital.gr (D.A.); korais8@yahoo.gr (C.H.); m.nikolaou@flemig-hospital.gr (M.N.); 2 Department of Internal Medicine, Amalia Flemig General Hospital, 15127 Melissia, Greece; kreouzi.m@live.unic.ac.cy; 3 Department of Cardiology, Medical School, National and Kapodistrian University of Athens, Attikon University General Hospital, 12462 Athens, Greece; lrallidis@med.uoa.gr; 4 1st Department of Cardiology, School of Medicine, National and Kapodistrian University of Athens, Hippokration General Hospital, 11527 Athens, Greece; vkkatsi@yahoo.gr (V.K.); stef6arch@yahoo.com (S.A.); 5 Department of Public Health Policy, School of Public Health, University of West Attica, 11521 Athens, Greece; esimou@uniwa.gr; 6 Department of Cardiology, Tzaneio General Hospital, 18536 Piraeus, Greece; kardiologiki@tzaneio.gov.gr; 7 First Department of Cardiology, National and Kapodistrian University of Athens, Hippocration General Hospital, 11527 Athens, Greece; ktoutouz@gmail.com

**Keywords:** obesity, inflammatory markers, ageing, immune system, inflammaging, elderly population

## Abstract

Obesity in elderly individuals is associated with increased levels of inflammatory biomarkers, indicating a state of chronic low-grade inflammation, which has been recently termed as adipaging. Several studies have demonstrated this relationship: overweight and obese middle-aged and elderly individuals show elevated levels of inflammatory markers like CXCL-16, IL-6, and adipokines compared to normal weight counterparts. These markers positively correlate with anthropometric parameters indicating increased cardiovascular risk. C-reactive protein (CRP) and fibrinogen levels increase progressively with higher obesity classes in the general population, including the elderly. For instance, CRP levels nearly double with each increase in weight class compared to normal weight individuals. Additionally, the presence of obesity-related comorbidities like hypertension or diabetes further elevates these inflammatory markers. In conclusion, obesity in the elderly is characterized by elevated levels of various inflammatory biomarkers, reflecting a state of chronic low-grade inflammation. This inflammatory state may contribute to the development of obesity-related comorbidities. The clarification of the complementary or independent role of these biomarkers in aging and obesity could lead to targeted therapeutic interventions in this vulnerable population group.

## 1. Introduction

Obesity is an emerging worldwide problem. According to the WHO report, in 2022, one in eight people in the world were living with obesity, meaning 16% of adults [1]. Its presence leads to the development of chronic diseases, including, among others, metabolic syndrome, cardiovascular disease, diabetes mellitus type 2, stroke, and non-alcoholic fatty liver disease. The aging population has multiple comorbidities, which will have a substantial impact on public health in the future [2]. By the late 2070s, the number of people aged 65 years or older globally is estimated to exceed 2.2 billion, outweighing the number of children (under age 18). By the mid-2030s, it is estimated that there will be 265 million people aged 80 years or older, more than the number of infants (1 year of age or less) [2]. The co-existence of obesity and aging is a matter of intense research, and various aspects of these two factors are addressed by researchers.

The phenomenon, known as the “obesity paradox,” with improved short- and mid-term survival rates in overweight versus better long-term survival outcomes in normal weight individuals, has also been studied as it was initially attributed to BMI’s inability to reflect fat distribution [3]. This paradox is more pronounced in people with chronic illnesses, such as cancer, heart failure, or kidney disease, and it seems to be caused by the lipolytic effect of cachexia. In other words, the detrimental effect of obesity is not linear, but also is a matter of the general status of the patient and other factors, such as the distribution of adipose tissue, which seems to play a role in the interpretation of obesity impact in the elderly population. This paradox is thus interrelated to sarcopenic obesity which occurs in 10% of older adults [4] and negatively affects quality of life and overall outcomes in the elderly population. It consists of obesity and loss of muscle mass and muscle function, whereas adipose tissue mass is increased. It is an entity that deserves the special attention of physicians when examining older patients [5].

Obesity reflects a state of chronic low-grade inflammation consisting of adipose tissue dysfunction, immune cell activation, and inflammatory signaling pathways [6], a state that is evident also in the obese elderly population [7]. 

On the other hand, conditions and comorbidities of obesity reflect those of aging and its related diseases. Aging and obesity exhibit analogous phenotypes that also include enhanced systemic inflammation [8]. Obesity and aging have been described as two sides of the same coin [9], whereas the term “adipaging” has also been used to show the common pathways of these two situations, resulting in a dysfunctional adipose tissue [10]. In this narrative review, we try to outline the obesity-related circulating or tissue inflammatory biomarkers in the elderly population and their possible clinical roles.

## 2. Methodology

A thorough analysis of the literature was carried out to find relevant studies published from July 2000 to September 2025. Electronic databases such as PubMed and Embase were used in the search. To guarantee comprehensive coverage of the relevant literature, the search method combined regulated vocabulary, such as Medical Subject Headings (MeSH), with free-text terms. To systematically integrate search phrases and refine the search process, Boolean operators (AND, OR) were used. “Obesity”, “inflammatory biomarkers”, “aging”, “immune system”, “inflammatory aging”, and “elderly population” were among the key terms. To gather further pertinent studies that were missed by the first database search, the reference lists of each article were also manually examined. To ensure agreement during the selection procedure, any differences in the eligibility assessment were settled by discussion among the investigators.

## 3. Changes in Adipose Tissue During Aging

The aging process is a lifelong process with a gradual decline in (pre-)adipocyte function over time [11]. Chronic unresolved inflammation in the adipose tissue alters immune cell profiles and tissue homeostasis [12]. These changes are not the same between the different types and depots of adipose tissue, nor are they the same throughout life. Human adipose tissue (AT) is categorized into three primary types: white adipose tissue (WAT), which is mainly involved in energy storage; brown adipose tissue (BAT), which plays a role in non-shivering thermogenesis; and beige adipose tissue, which contains adipocytes with characteristics that are intermediate between white and brown fat. These tissue types are distributed throughout the human body [13].

Concerning WAT, it consists of subcutaneous adipose tissue (SAT), which lies in the hypodermis, and includes the subcutaneous thoracic, abdominal, gluteal, or femoral adipose tissue. Visceral adipose tissue (VAT), which is the adipose tissue located inside the abdominal or thoracic cavity, between the viscera, can be sub-classified into thoracic adipose tissue (ThAT) (pericardial, non-pericardial, epicardial, and perivascular) and abdominal adipose tissue (intraperitoneal, retroperitoneal) [14]. SAT decreases with age, whereas abdominal and pericardial VAT increase with age [15]. Visceral AT (“metabolically unhealthy”) is considered metabolically harmful and associated with increased risk, whereas subcutaneous AT (“metabolically healthy”) appears neutral or even beneficial.

An enhanced collection of senescent cells has been found in the visceral fat of obese individuals, which is an important source of inflammatory cytokines [16]. WAT is known to secrete multiple pro-inflammatory biomarkers, such as visfatin, leptin, chemerin, resistin, and CRP, cytokines such as IL-6, and anti-inflammatory biomarkers, such as adiponectin. A chronic condition of subclinical inflammation in obesity has been shown to shift immune cells from an anti-inflammatory to a pro-inflammatory state, among others, from B1 (anti-inflammatory) cells to B2 (pro-inflammatory) cells [17]. The two subsets of B cells perform nearly opposing functions in adipose tissue; in particular, B-1 cells provide an early, innate-like defense and act as a brake on inflammation through natural IgM and IL-10 production, whereas B-2 cells drive a later, adaptive immune response that, under conditions like high-fat diets or aging, can lead to heightened inflammation through the triggering or enhancement of the production of inflammatory cytokines such as IL-6 and TNF-α. This further propels the inflammatory cycle in adipose tissue and metabolic dysfunction, leading to conditions such as insulin resistance and atherosclerosis [17].

Brown adipose tissue (BAT) declines with aging, especially in male adults, with the interscapular areas being the first to decline [18]. Other areas of BAT are the supraclavicular areas, which are more active in females, but the detection of BAT in humans is generally difficult [19]. Also, beige adipose tissue becomes dysfunctional with aging due to several mechanisms, such as chronic inflammation and senescence of different adipose cell compartments [19]. Their role in local inflammation as compared to WAT is less pronounced, although enhanced production of TNF-a and MCP-1 is caused due to obesity-related metabolic derangement [20]. The beige-like epicardial adipose tissue (adipose tissue between the myocardium and the visceral side of the pericardium) changes function in obesity. From being a protective thermogenetic tissue to the myocardium, it becomes inflammatory, secreting pro-inflammatory cytokines (e.g., MCP-1, TNFa, and IL-1b) into the adjacent myocardium and coronary arteries, with a possible damaging role in the coronary vasculature [21].

**Figure 1:**
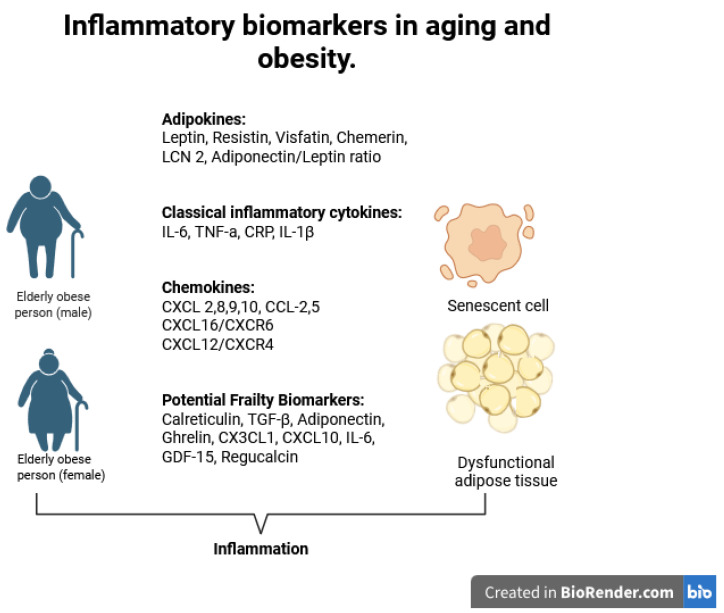
Inflammatory biomarkers in aging and obesity.

Overall, there is a variety of common and novel inflammatory biomarkers [22]. Present studies have focused on the description of these markers in obesity [23] or in aging [24]. In this narrative review, a description of the existing evidence of obesity-related inflammatory biomarkers in the elderly population follows, trying to highlight their possible clinical value in the daily clinical practice (Figure 1). Also, a summary of the biomarkers and their role in obesity and aging and their clinical implications can be seen in Table 1.

## 4. Plasma Visfatin Levels in Obesity and Aging

Visfatin is a protein mainly secreted from VAT not only by adipocytes, but also by macrophages infiltrating adipose tissue [25]. Its aggravating effects on atherosclerosis and metabolic syndrome have been studied in vitro and ex vivo, but some beneficial effects could also be present depending on the clinical scenario [97]. An affinity of this protein for the insulin receptor has been shown in animal models by binding to the receptor at a different, from insulin, linking site [98], thus implying a possible role of this protein in abnormal glucose metabolism [26], which was not confirmed in clinical studies [27]. More specifically, Pagano et al. [27] concluded that in adults of a similar age free of diabetes, only waist circumference was associated with the HOMA insulin resistance index, suggesting that differences can be due to sample differences and methodology. Conflicting evidence exists regarding whether visfatin levels are related to the amount of VAT. Higher levels of mRNA visfatin have been shown in the VAT in obese versus lean subjects, which was also positively related to BMI [27], but visfatin mRNA expression was lower in the SAT of obese versus lean subjects in the same study, a result that implies a difference in expression depending on the type of AT studied. Moreover Jurdana M et al. [28] have shown significantly higher baseline levels of fasting visfatin in overweight versus normal weight subjects and confirmed statistically that physical fitness was the best significant predictor of the baseline visfatin concentration in male participants Alanine, omega-3 fatty acid intake, and C-reactive protein levels were significant predictors of baseline visfatin concentrations in females, suggesting a gender difference in the expression of this adipokine. In morbidly obese adults, plasma visfatin levels were significantly increased compared to lean subjects [29] and its levels decreased significantly after standardized laparoscopic adjustable gastric banding. After both gastric banding and biliopancreatic diversion surgery in morbidly obese adults with or without glucose metabolic abnormalities, visfatin levels increased, possibly through changes in hormone secretion from the GI tract. This was the result of the combined surgery leading not only to restriction in food intake, but also to malabsorption of nutrients [30], thus showing a difference in the effect of the surgery type in the visfatin levels.

Concerning the correlation of visfatin with anthropometric parameters, again, various studies have shown divergent results. In a study of obese adults (BMI > 30) without a previous diagnosis of abnormal glucose metabolism, the only negative correlation was found with the waist to hip ratio (WHR) [26], suggesting an association with the gynoid pattern of adipose tissue distribution, but the researchers commented that their study included 70% women who usually exhibit the gynoid adipose tissue pattern obesity. In another study [31], plasma visfatin levels and visfatin mRNA expression were measured in 189 adults with a wide range of obesity, body fat distribution, insulin sensitivity, and glucose tolerance. The authors conclude that visfatin plasma concentration correlates positively with the visceral visfatin mRNA expression, BMI, and percent body fat, but not with WHR. These results could be explained based on the heterogeneity of subjects of the population studied, as well as on the high variability of visfatin expression in VAT and SAT.

Few studies are addressing the relationship between age and plasma visfatin levels, but their results seem consistent. Kaminska et al. [26] showed a decrease in plasma visfatin levels with age and glycated hemoglobin levels in 68 obese adults without a previous diagnosis of abnormal glucose metabolism. In another study by de Luis et al., plasma visfatin levels were also found to be inversely correlated with age in an interventional longitudinal study analyzing a population of 80 obese nondiabetic outpatients, showing a steady decrease in levels for each year of age [32]. Altogether, visfatin is an inflammatory adipokine with a plethora of studies relating it with obesity. Its cardiometabolic effects in the elderly require further studies, since it shows a gradual decrease with age in obese nondiabetic subjects.

## 5. Leptin

Leptin is a 16 kDa protein containing 160 amino acids that is responsible for food intake, body mass, reproductive functioning, and fetal development [33]. It is primarily produced in white adipose tissue and also in smaller concentrations in brown adipose tissue, placenta, stomach, fetal tissue, bone marrow, muscles, teeth, and the brain. Hyperleptinemia is found in obesity as its secretion is proportional to body mass and nutritional status. Its secretion is more pronounced in the subcutaneous rather than in the visceral adipose tissue [34]. There is a feedback loop explaining the increased production of multiple inflammatory cytokines (IL-6, IL-12, TNF-α) from leptin and the increased expression of leptin from others (e.g., IL-1, TNF-α), leading to a chronic inflammatory state due to obesity [35]. There is also evidence that obesity impairs the effects of leptin, leading to resistance that also involves its receptor’s function, LEP-R [34]. This also explains the diminished effects of exogenous leptin administration and the need for leptin sensitizers [33] as leptin resistance occurs due to the leptin’s inability to reach the target cells, reduced Lep-R expression, or disturbed Lep-R signaling [36,37].

Elevated levels of circulating leptin in older adults primarily result from increased fat mass when compared to younger adults. Another reason for this elevation has been suggested to be due to decreased leptin responsiveness with advancing age as a result of the impaired signaling of hypothalamic leptin receptors, a phenomenon that has been observed in aged rats [38]. In older humans, a reduction in the expression of the short form of the leptin receptor (LepRa) in peripheral blood monocytes has been documented. LepRa is recognized for its role in transporting leptin across the blood–brain barrier [39]. Whether aging reduces hypothalamic leptin responsiveness in humans remains to be determined, as it is linearly correlated with total body fat and BMI. In line with the above evidence, Kohala et al. [40] have shown that plasma levels of leptin are further increased in subjects with sarcopenic visceral obesity compared to those with either sarcopenia or visceral obesity alone. These findings suggest that leptin may link visceral obesity and sarcopenia, possibly through the reduction in Lep-R due to muscle loss, leading to the amplification of leptin levels.

It has been shown that the adiponectin/leptin (AL) ratio is a better diagnostic marker for classifying subjects with metabolic syndrome than leptin or adiponectin alone [41]. Increased adiponectin is associated with increased mortality in the elderly, perhaps related to production by the vascular system in atherosclerosis [42,43]. An interesting article by Senkus et al. [44] evaluated the AL ratio in elderly (over 65 years) obese adults at baseline and 12 months after lifestyle and diet intervention and concluded that AL ratio is an important marker in AT dysfunction and cardiometabolic risk in older adults. At baseline, a lower AL ratio was linked to higher BMI, waist circumference, adiposity, and insulin levels. In women, the AL ratio also correlated with HDL cholesterol and inflammatory markers (hsCRP, IL-6). After 12 months, both the weight maintenance and intentional weight loss groups showed significantly elevated AL ratio, mostly in the intentional weight loss group. Improvements in AL ratio were associated with reductions in abdominal fat and trunk fat. Thus, monitoring the AL ratio may identify older adults at risk for cardiometabolic diseases and guide interventions.

## 6. Retinol Binding Protein 4 (RBP4) in Obesity and Aging

RBP4 is recognized as a negative acute inflammatory reactant and as a novel adipokine, whose levels increase in insulin-resistant conditions, such as obesity, metabolic syndrome, type 2 diabetes mellitus [45], as well as cardiovascular diseases [46]. It is also produced in the liver and in macrophages [45]. In elderly patients, as those studied in the PIVUS study (Prospective Investigation of the Vasculature in Uppsala Seniors), circulating RBP4 concentrations were inversely associated with intima media and plaque echogenicity in carotid arteries, implying its role in the development of atherosclerosis [47]. RBP4 contributes to the advancement of insulin resistance via immune and inflammatory processes in adipose and vascular tissues [48].

According to a study by Gavi S et al. [51] comparing a young and an elderly small group of adults, in the elderly subjects, there was no correlation between RBP4 levels and insulin sensitivity, percent trunk fat, triglycerides, and low-density lipoprotein, and its levels in the elderly seem to be independent of central adiposity [49]. In one other study with a small group of obese adults over 70 years of age vs. non-obese elderly adults, RBP4 was found to be associated with the HOMA index only in the obese group [50]. Overall, a correlation of RBP4 with insulin resistance in obesity and aging cannot be ruled out, but further larger studies are needed to focus on this subpopulation.

## 7. Chemerin in Obesity and Aging

Chemerin is a recently discovered adipose tissue-specific adipokine that has a pivotal function in the differentiation and development of adipocytes, in addition to glucose and lipid metabolism [51]. Elevated levels of chemerin were detected in overweight individuals, in prediabetic states, and in lean, overweight, and obese individuals with T2DM [52].

In elderly patients with type 2 DM, circulating chemerin levels are elevated and are independent of the length of disease and BMI, probably due to adipocyte dysfunction enhancement with aging [53]. This study did not use other anthropometric measurements, and BMI does not show fat distribution. Chemerin is directly associated with age but it is uncertain if elevated chemerin is a result of aging or of the higher concentration of visceral adipose tissue in the aging population [54]. So, further larger clinical trials of chemerin in obesity and aging are needed to clarify these associations.

## 8. Resistin in Obesity and Aging

Resistin was initially described as an adipokine in mice by Steppan et al. [55], linking obesity and diabetes. Its role as an adipokine has not been established in humans, where its primary site of production is mainly in monocytes and macrophages, and it is associated with an increased risk of atherosclerosis and a propensity for insulin resistance in elderly patients with a history of previous coronary interventions [56].

Resistin levels show an age-related increase. In elderly subjects, resistin is increased in all situations of inflammaging. Offspring of centenarians show lower rates of MetS, better glucose tolerance, and higher insulin sensitivity compared to age-matched controls. MetS-affected offspring of centenarians experience fewer cardiovascular events, lower rates of hypertension and hypercholesterolemia, and lower resistin levels compared to those without a family history of longevity. No significant differences were found in other inflammatory markers or adipokines between groups. In this context, heredity appears to play an important role in regulating adipokines and maintaining metabolic health during aging [57]. Resistin could be a potential biomarker for estimating the risk of age-related chronic diseases.

## 9. Lipocalin 2 in Obesity and Aging

Lipocalin 2 (LCN2), also known as neutrophil gelatinase-associated lipocalin (NGAL) [58], has been studied in various conditions, such as inflammation, infection, and metabolic diseases. It is a 25 kDa glycoprotein derived from neutrophil granules and is a member of the lipocalin superfamily [59]. It is produced abundantly in adipocytes, particularly after preadipocytes mature, but is also secreted by other tissues, such as the liver and kidney tissues. Despite LCN2 levels increasing under a large number of inflammatory conditions, both the pro- and anti-inflammatory properties of this adipokine have been reported [60].

A study by Daoud et al. [61] examined the correlation between LCN2 and other adipokines in obese adults undergoing a 12-month lifestyle and diet-induced weight reduction, before and after the intervention, and found that LCN2 was positively correlated with resistin and adiponectin. These results imply a role for LCN2 in adipose tissue dysregulation and could recognize LCN2 as a biomarker for diagnosing obesity-associated metabolic disorders [62].

Increased LCN-2 expression is observed in the adipose tissue of obese individuals and is linked to obesity-related variables, including, among others, fasting glucose and the HOMA-IR index [63]. LCN-2 is induced by various pro- and anti-inflammatory cytokines and has been identified as pivotal in glucose homeostasis and insulin sensitivity. Its association with body fat mass and metabolic indices underlines its role in evaluating the severity of obesity [64].

In aging, the role of LCN2 is not yet clarified. In LCN2-deficient KO mice [65], lipocalin-2 deficiency protects mice from developing aging- and obesity-induced insulin resistance largely by modulating 12-lipoxygenase and TNF levels in adipose tissue. Conversely, ap2-promoter-driven LCN2 transgenic (Tg) mice and aged LCN2 Tg mice showed that overexpression of LCN2 in adipose tissue not only preserves adipose tissue function during aging but also promotes maintenance of glucose tolerance, decreases dyslipidemia, and prevents liver lipid accumulation and steatosis [64]. A cross-sectional study of adults without major disease showed that LCN2 increased across the lifespan [65]. LCN2 also increased with higher BMI and decreased with higher aerobic fitness, independent of gender [65]. Future studies could give insight into the clinical implications of LCN2 variations across different clinical conditions and examine the potential role of LCN2 as a therapeutic target for the prevention of age-related disease risk or severity.

## 10. TNF-a, IL-1β, and IL-6 in Obesity and Aging

The aging immune system undergoes quantitative and qualitative changes, leading to a reduced ability to fight infections and increased susceptibility to cancer and autoimmune disorders. Key features include altered T-cell populations, a diminished ability to recognize diverse antigens, reduced effector functions, and a chronic, low-grade inflammatory state [66], as already mentioned before. The role of circulating pro-inflammatory cytokines (interleukin (IL)-6, tumor necrosis factor (TNF), or IL-1b) as a risk factor in cardiovascular and neurodegenerative diseases is well established, as well as its association with sarcopenia and frailties [67]. Many studies have examined the relation of these cytokines with age [68,69] though a clear association cannot be established due to differences in the characteristics of the subpopulations studied. Nevertheless, in a small population of a healthy non-obese adults study, a trend in the increase in TNF-a levels with age was found, whereas for IL-6 levels, there was a positive correlation [70]. The role of these cytokines in aging seems to be dichotomous since studies have shown that cytokines, like IL-1β, IL-6, and TNF, positively regulate macroautophagy, mitochondrial function, anti-tumor immune responses, and skeletal muscle biogenesis, possibly contributing to longevity. Contradictorily, there is also a detrimental and antagonistic role of these cytokines, including the induction of sarcopenia, tissue damage, and promotion of tumorigenesis [71].

Ghanemi et al. [72] have shown shared patterns in the aging and obesity process in cellular, molecular, and epigenetic pathways that impact health outcomes. In obese older adults, higher levels of adiposity are associated with higher blood levels of inflammatory markers, such as interleukin (IL)-1 receptor antagonist (IL-1RA), IL-6, and TNF-α [73]. These pro-inflammatory cytokines inhibit preadipocyte differentiation and maturation, and promote adipocyte aging [16]. IL-1β, IL-6, and TNF-α secreted by adipose tissue macrophages reduce PPAR-γ expression, an important transcription factor that induces adipogenesis [73], while finally there is a shift in macrophage migration and expression resulting in enhanced expression of M1 macrophages (classically activated CD40+ and/or CD11c+), a pro-inflammatory macrophage phenotype, in aged obese individuals, which is formed with aberrant adipose tissue [73].

Concerning the role of IL-6 in aging and obesity, a positive and significant association between serum IL-6 and visceral fat mass has been shown in a small cohort of 77 patients aged ≥ 65 years old [74]. Furthermore, the authors have exhibited that moderately increased IL-6 levels during the aging process inhibit PKA/HSL-mediated lipolysis in KO mice and consequently lead to an increase in lipid accumulation in visceral adipose tissue [74]. The role of IL-6 in insulin resistance remains controversial, although elevated levels of IL-6 have been linked to the development of type 2 diabetes [75]. The different actions of IL-6 on insulin signaling may be due to its divergent actions in variable organs (liver versus muscle) or the different sources of IL-6 (muscle versus fat) [76].

Altogether, these three inflammatory biomarkers are elevated in obesity and aging, promoting adipose tissue dysfunction, and are implicated in the chronic state of low-grade inflammation.

## 11. CRP Levels Under the Scope of Obesity and Aging

The aging process is associated with senescence of the immune system [77], particularly with T-lymphocyte dysfunction [78], as well as with a recently described phenotype called SCAP (senescence-associated secretory phenotype) [24]. It is known that in healthy normal weight subjects, abdominal obesity is associated with hs-CRP, irrespective of age and BMI [79]. A value of hs-CRP over 3 mg/dL is linked to elevated cardiovascular risk [80], and the proportion of people with elevated hs-CRP is significantly higher in those with abdominal obesity than in control subjects, in addition to a higher BMI [79]. Concerning the trend of CRP levels in elderly people of the general population, Hutchinson et al. have shown that there is an increase (approximately a doubling of the median CRP levels in the oldest from 1 to 2 mg/dL) with higher values in the female subjects [81]. In obese older adults, higher levels of adiposity are associated with higher blood levels of the acute phase reactant, C-reactive protein, especially in female nonagenarians [72], making it a potential add-on biomarker for cardiovascular risk stratification.

CRP is an established biomarker of the IL-1β/IL-6/CRP axis [82]. Although its genetic variations have not been directly linked to CVD risk [80], newer genetic studies have found that less common recessive genotypes of two single nucleotide polymorphisms in the *CRP* gene (rs1800947 and rs11265263) were associated with significantly higher mortality risk in heart failure patients [83].

## 12. The Role of Chemokines in Obesity and Aging

Chemokines are signaling proteins that guide white blood cells to locations of injury or infection by initiating a process called chemotaxis. As a type of “cytokine”, chemokines bind to receptors on immune cells, stimulating them to move toward specific sites to fight inflammation. They have a pivotal role in the proper functioning of the immune system, collaborating to orchestrate immune responses by navigating the migration and activating immune cells [84]. Currently, more than 50 chemokines and 18 chemokine receptors, having various physiological and pathological properties, have been discovered, and their receptors can be common (shared receptors) but with divergent functions [85]. They are distinguished into inflammatory and homeostatic chemokines, the former being responsible for recruiting neutrophils and monocytes to sites of active inflammation, whereas the latter seem to have a more qualitative (highly specific) control on immune cells’ migration [85].

In obesity, pro-inflammatory cytokines and chemokines involved in the recruitment of immune cells to the adipose tissue from bone marrow are secreted so that consequently macrophage infiltration within the adipose tissue becomes one of the major sources of inflammation [85]. Adipocytes release pro-inflammatory biomarkers as well (cytokines, chemokines, and adipokines) and in larger amounts compared with immune cells. Another source of chemokine production in the adipose tissue is interferon-γ, the signature Th1 cytokine, which causes macrophages and T cells to release chemokines, which in turn recruit immune cells to the obese adipose tissue [86]. Despite extensive trials, scarce evidence has been found as to the differential expression of chemokines that account for the chronic inflammatory state in obesity.

In brief, in obesity, adipose tissue secretes several chemokines, including MCP-1 (also known as CCL2) and CCL5, which can trigger signaling pathways and result in inflammatory reactions and insulin resistance. These elements can likewise influence the management of obesity. Weight reduction has demonstrated a decrease in the serum levels of chemokine MCP-1 in obese males with metabolic syndrome and enhances indicators related to kidney damage [87]. The up-regulation of CCL2 results in an increased recruitment of macrophages into adipose tissue, worsening metabolic inflammation, though CCL2-deficient mice do not show decreased macrophage infiltration into the adipose tissue [88].

CXC chemokines (including CXCL16/CXCR6, CXCL10, and CXCL12/CXCR4) influence inflammation linked to obesity and affect immune cell migration, tumor development, and metabolic processes [89]. CXCL12, a chemokine secreted by adipocytes, attracts macrophages into adipose tissue, impairing insulin sensitivity [90]. On the other hand, the CXCL16/CXCR6 axis in adipocytes differentiated from human adipose-derived mesenchymal stem cells regulates macrophage polarization, making it a possible target as a modulator of immune response [89]. Patients with obesity have been noted to have increased circulating levels of the CXCL10 chemokine. These levels are associated with indicators of obesity, including BMI, waist circumference, and Homeostatic Model Assessment of Insulin Resistance (HOMA-IR) [85]. Increased CXCL10 levels might boost leukocyte attachment to endothelial cells, leading to dysfunctional endothelial activity. This mechanism may play a role in the onset of insulin resistance and cardiovascular issues associated with obesity [91].

Concerning aging, overweight and obese middle-aged and elderly individuals show elevated levels of the inflammatory chemokine CXCL-16 compared to normal-weight counterparts [92]. This marker positively correlates with anthropometric parameters, indicating increased cardiovascular risk. CXCL16 could potentially serve as an early marker for the transition from normal weight to overweight in this age group [92]. Bonfante et al. have shown that serum levels of CCL2, CXCL8, CXCL9, and CXCL10 increase with aging, considering them as potential aging biomarkers [93]. Also, Inadera et al. elicited a profound age-dependent increase in CCL2 levels both in males and females, most possibly due to the correlation of CCL2 levels with atherosclerotic burden, although no difference was found in patients with coronary artery disease compared to healthy age-matched controls. Furthermore, CXCL9 has a distinct role in cardiovascular aging and vascular dysfunction, which is associated with cellular senescence and arterial stiffness. Its silencing reverses these phenotypes, and most probably it could serve as an early indicator of aging [94]. Cardoso et al. have highlighted CX3CL1 and CXCL10, along with other factors, such as IL-6, GDF15, regucalcin, calreticulin, TGF-β, adiponectin, and ghrelin, as potential frailty biomarkers [95]. In their review, Chaudhary et al. describe recent findings about the role of chemokines in the aging process of different organs and underline the crucial importance of targeting these mechanisms in order to delay and/or modulate the aging process and prevent age-related diseases [96].

## 13. Conclusions

Obesity in the elderly is associated with a chronic, low-grade inflammatory state, often referred to as “adipaging”, whereas “inflammaging” also includes other causes of chronic inflammation during aging. This inflammation is reflected by elevated levels of various inflammatory biomarkers (such as visfatin, leptin, chemerin, resistin, lipocalin 2, TNF-α, IL-1β, IL-6, CRP, and several chemokines). These biomarkers are linked to the development and progression of obesity-related comorbidities (e.g., cardiovascular disease, diabetes, metabolic syndrome) in older adults. The interplay between obesity and aging leads to dysfunctional adipose tissue, which further enhances systemic inflammation and increases vulnerability to disease. There is evidence that some biomarkers (e.g., CRP, IL-6, CXCL16) increase with both age and adiposity and may serve as early indicators of risk of frailty in the elderly population. Understanding the complementary or independent roles of these biomarkers could help in developing targeted therapies to improve health outcomes in this rapidly growing population. More research is needed to clarify the mechanisms and to identify which biomarkers are most clinically useful for risk stratification and intervention in elderly obese individuals.

## Figures and Tables

**Table 1:** Inflammatory biomarkers linked to obesity in older adults.

Biomarker	Site of Production	Association with Obesity and Aging	Clinical Implications	Ref.
Visfatin	Mainly VAT (adipocytes and macrophages)	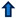 in obesity levels; 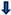 with age in obese nondiabetic subjects; associated with VAT, BMI, and body fat; conflicting data on correlation with fat distribution.	Potential marker for cardiometabolic risk; role in elderly requires further study.	[25,26,27,28,29,30,31,32]
Leptin	Primarily WAT; also, BAT, placenta, other tissues	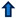 in obesity and with increased fat mass in elderly; leptin resistance common in obesity and aging; higher in sarcopenic visceral obesity; AL ratio is a better marker for metabolic syndrome.	Marker for MetS and cardiometabolic risk; AL ratio useful for risk stratification and monitoring interventions.	[33,34,35,36,37,38,39,40,41,42,43,44]
RBP4	Adipose tissue, liver, macrophages	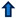 in IR states (obesity, metabolic syndrome, T2DM); in elderly, not always correlated with adiposity; may be associated with atherosclerosis and IR in obese elderly.	Potential marker for atherosclerosis and IR; more studies needed in elderly.	[45,46,47,48,49,50]
Chemerin	Adipose tissue	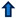 in overweight, prediabetes, T2DM; in elderly, levels increase with age and are independent of BMI; may reflect adipocyte dysfunction with aging.	Possible marker for metabolic dysfunction in elderly; further studies needed for clinical use.	[51,52,53,54]
Resistin	Monocytes, macrophages (humans)	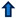 with age and in inflammaging; associated with insulin resistance and atherosclerosis in elderly; lower in offspring of centenarians with better metabolic health.	Potential biomarker for age-related chronic disease risk and metabolic health.	[55,56,57]
Lipocalin 2 (LCN2)	Adipocytes, neutrophils, liver, kidney	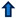 in obesity and with age; correlates with resistin and adiponectin; associated with body fat, glucose, HOMA-IR; role in aging not fully clarified.	May serve as a biomarker for obesity-related metabolic disorders; possible therapeutic target for age-related disease prevention.	[58,59,60,61,62,63,64,65]
TNF-α, IL-1β, IL-6	Adipose tissue macrophages, immune cells	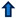 in obesity and aging; promote adipose dysfunction, sarcopenia, frailty; IL-6 correlates with visceral fat in elderly; dual roles (pro- and anti-inflammatory effects).	Risk factors for CVD, diabetes, sarcopenia, frailty; potential targets for anti-inflammatory interventions.	[66,67,68,69,70,71,72,73,74,75,76,77]
CRP	Liver (in response to IL-6 and IL-1β)	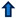 with adiposity and age; higher in elderly, especially women; associated with CV risk; genetic variants linked to mortality in heart failure patients.	Established biomarker for inflammation and CVD risk; useful for risk stratification in elderly obese individuals.	[78,79,80,81,82,83,84]
Chemokines (e.g., CXCL16, CCL2, CXCL10, CXCL9)	Adipose tissue, immune cells, endothelial cells	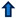 in obesity and aging; associated with increased CVD risk, insulin resistance, and frailty; some (e.g., CXCL16, CCL2) increase with age and adiposity.	Potential early markers for frailty, CVD, and metabolic dysfunction; possible therapeutic targets.	[85,86,87,88,89,90,91,92,93,94,95,96]

## Data Availability

Not applicable.

## Abbreviations

The following abbreviations are used in this manuscript:

AL	Adiponectin/Leptin
BMI	Body mass index
CRP	C-reactive protein
GI tract	Gastrointestinal tract
HOMA	Homeostasis model assessment
IL	Interleukin
LCN2	Lipocalin 2
MetS	Metabolic syndrome
RBP4	Retinol binding protein 4
SAT	Subcutaneous adipose tissue
VAT	Visceral adipose tissue
VSF	Visfatin
WC	Waist circumference
